# Antibiotics induced intestinal tight junction barrier dysfunction is associated with microbiota dysbiosis, activated NLRP3 inflammasome and autophagy

**DOI:** 10.1371/journal.pone.0218384

**Published:** 2019-06-18

**Authors:** Yanhai Feng, Yalan Huang, Yu Wang, Pei Wang, Huapei Song, Fengjun Wang

**Affiliations:** 1 State Key Laboratory of Trauma, Burns, and Combined Injury, Institute of Burn Research, Southwest Hospital, Third Military Medical University (Army Medical University), Chongqing, China; 2 Department of Military Nursing, School of Nursing, Third Military Medical University (Army Medical University), Chongqing, China; 3 Department of Gastroenterology, Southwest Hospital, Third Military Medical University (Army Medical University), Chongqing, China; University of Chicago Medical Center, UNITED STATES

## Abstract

Tight junction barrier is critical to intestinal homeostasis. Applying antibiotics to treat infections is common in clinical practice, which may affect intestinal microbiota. Intestinal microbiota dysbiosis is involved in the occurrence of some gastrointestinal diseases. Therefore, this study was aimed to investigate the influence of antibiotics on intestinal tight junction barrier and the possible underlying mechanisms. Healthy adult female C57BL/6 mice were treated with a broad-spectrum antibiotic cocktail for 14 days. 16S rDNA Illumina sequencing and headspace gas chromatography-mass spectrometry (HS-GC/MS) were respectively used to analyze microbial community and to detect short-chain fatty acids (SCFAs) contents. *In vivo* intestinal paracellular permeability to fluorescein isothiocyanate-dextran (FITC-dextran) was measured. Protein expression was determined by immunoblotting. Immunofluoresence was applied to observe the distributions of ZO-1, LC3B and ASC. Antibiotics remarkably altered intestinal microbiota composition in healthy mice, accompanying reduced SCFAs’ concentrations. In addition, the intestinal tight junction barrier was disrupted by antibiotic treatment, as evidenced by increased intestinal paracellular permeability to FITC-dextran, decreased tight junction protein expressions, and disrupted ZO-1 morphology. Furthermore, NLRP3 inflammasome and autophagy were activated by antibiotic treatment. In conclusion, intestinal epithelial tight junction barrier dysfunction induced by antibiotics is associated with intestinal microbiota dysbiosis, activated NLRP3 inflammasome and autophagy in mice.

## Introduction

A primary function of intestinal epithelium is to form a biological barrier which prevents luminal antigens or pathogens from entering into mucosa and contacting with immune system, resulting in intestinal homeostasis [1,2]. Tight junction and its associated proteins, including zonula occludens (ZO), occludin and claudins, are the important factors which contribute to the formation of intestinal tight junction barrier [1–3]. However, disrupted intestinal epithelial tight junction barrier is compromised in a variety of diseases, such as inflammatory bowel disease, intestinal ischemia/reperfusion injury, shock, and severe burns [1–6]. To date, the precise mechanisms of intestinal tight junction barrier dysfunction are still not fully elucidated, and need to be further investigated.

Various microorganisms, including bacteria, archaea, viruses and various unknown eukaryotes, inhabit in human gastrointestinal tract, and have been named intestinal microbiota [7]. It has been reported that intestinal microbiota contributes to intestinal epithelial barrier improvement, immune system development, nutrients absorption and pathogens colonization restriction [8]. Therefore, intestinal microbiota is believed to play a critical role in maintaining intestinal homeostasis. However, the intestinal microbiota can be altered by many factors and diseases, including stress, intestinal ischemia/reperfusion, infection, dietary changes, and antibiotics [9–12], resulting in the imbalance of intestinal homeostasis.

As an invaluable weapon to fight infectious diseases, antibiotics have been used to treat bacterial infections for many years. Although antibiotics brings significant benefits for patient, it causes some grievous adverse consequences, among which multidrug-resistant pathogen infection might be the most serious one [13]. In fact, antibiotics affects not only the target microorganism but also the microbial communities, especially the intestinal microbiota. It has been documented that antibiotics induced long-lasting changes in intestinal microbiota, which correlates with diseases [14]. Although antibiotics have a remarkable impact on intestinal microbiota, whether they affect the intestinal tight junction barrier remains unclear.

In this study, we aimed to determine the effect of antibiotics on intestinal barrier and the possible mechanisms in C57BL/6 mice. It is revealed that antibiotics disrupted intestinal barrier function, impaired intestinal microbiota homeostasis, decreased SCFAs contents, activated NLRP3 inflammasome and autophagy. Thus, it is suggested that antibiotics disrupt intestinal barrier dysfunction, which is associated with intestinal microbiota dysbiosis and activated NLRP3 inflammasome and autophagy.

## Materials and methods

### Ethics statement

All experimental animal manipulations were conducted in accordance with the Animal Care and Use Committee of Daping Hospital, Army Medical University (Third Military Medical University) with the animal license SCXK(J)2007-017, and all the protocols were permitted by the Medical and Ethics Committee of Southwest Hospital, Army Medical University (Third Military Medical University), Chongqing, China. The project license number is NSFC81772081.

### Animals and antibiotics treatment

Healthy adult female C57BL/6 mice weighing 18-22g were used in this study. Mice were housed in wire-bottomed, wire-lid cages, allowed access to chow and water *ad libitum*, and acclimatized in a temperature-controlled room (25±2°C) with 12-hour light and dark cycles for 1 week before experiments. To induce intestinal microbiota dysbiosis, healthy mice were treated with broad-spectrum antibiotics as described previously [15]. Briefly, broad-spectrum antibiotics cocktail, consisting ampicillin (1g/L, Sigma), neomycin sulfate (1g/L, Amresco), metronidazole (1g/L, Sigma) and vancomycin (0.5g/L, Amresco), was administered to mice *ad libitum* via drinking water for 1, 3, 5, 7 or 14 days respectively. At each time point, mice were anesthetized for intestinal paracellular permeability assay, and then sacrificed for sample collections. Samples were used for the following experiments.

### *In vivo* intestinal paracellular permeability assay

According to the methods we described previously [16–18], intestinal paracellular permeability was determined by measuring the appearance of a maker in blood, 4.4kDa fluorescein isothiocyanate-labeled dextran (FITC-dextran) (Sigma, St. Louis, MO). Briefly, a laparotomy was carried out under anesthesia before the mice were sacrificed at the end of experiment. A 5-cm segment of ileum was dissociated beginning 5cm proximal to the cecum, with well-protected superior mesenteric vessels. The bilateral end of the isolated ileum was ligated with 2–0 silk suture to prevent the leakage of FITC-dextran. Injecting 0.1ml of 20mg/ml FITC-dextran, which was dissolved in 0.1mol/L phosphate buffer saline (PBS, PH 7.2), into the lumen. After this procedure, the laparotomy was closed. After 30 min, blood samples were taken, and centrifuged at 3000g for 10 min. The plasma was collected and diluted at 1:10 with 0.1mol/L PBS. Then the fluorescence intensity of the diluted plasma was measured by using a microplate reader (Varioskan Flash, Thermo Electron Corporation, Vantaa, Finland) with an excitation wavelength of 480nm and an emission wavelength of 520nm. The plasma FITC-dextran concentrations were calculated from standard curves generated by serial dilution of FITC-dextran in PBS.

### Immunofluorescent staining, microscopy, and image analysis

Frozen sections of ileal tissue were fixed with 4% paraformaldehyde for 10 min at room temperature, then washed thrice with PBS, permeabilized in 0.1% Triton X-100 in PBS for 5 min at room temperature. After washing with PBS, the nonspecific binding sites were blocked with 5% normal goat serum in PBS for 30 min. Then, the sections were incubated with monoclonal rabbit antibody against ZO-1 (1:200, Invitrogen), ASC (1:100, Cell Signaling), LC3B (1:100, Sigma) at 4°C overnight. After washing thrice in PBS, sections were incubated with secondary Alexa Fluor 594 conjugated goat anti-rabbit IgG antibody (1:200, Invitrogen) and DAPI (1:1000, Sigma) for 1 hour at room temperature. After washing thrice in PBS, sections were mounted using Slowfade reagents (Invitrogen). Images were obtained using a TCS SP5 laser confocal microscopy (Leica, Germany).

### Immunoblotting analyses

An appropriate ileal segment was taken to collect mucosa by a glass slide after the mice were sacrificed. The collected mucosa was homogenized with 10 volumes of ice-cold lysis buffer containing 100mM PMSF, protease and phosphatase inhibitor cocktails (KeyGEN BioTECH), followed by a brief sonication with a sonicator (Tomy Seiko, Tokyo, Japan). Thereafter, the homogenates were centrifuged at 4°C, 10000 rpm for 15 min, and the supernatants were collected to determine protein concentration using *RC DC* kit (Bio-Rad, Hercules, CA). Equal amounts of total protein extracted from the ileal mucosa were fractionated on 8%, 10% and 12% sodium dodecyl sulfate-polyacrylamide gel electrophoresis (SDS-PAGE) gel to analyze the expressions of tight junction proteins (ZO-1, occludin and claudin-1), autophagy proteins (Atg5, p62 and LC3) and NLRP3 inflammasome proteins (NLRP3, ASC, caspase 1, cleaved caspase 1, IL-1β, cleaved IL-1β and IL-18). Then the proteins were transferred onto polyvinylidene difluoride (PDVF) membrane (Millipore, Bedford, MA). After complete transfer, membranes were blocked with 5% nonfat milk dissolved in TBST buffer for 60 min at room temperature. After blocking, membranes were incubated with antibodies specific for ZO-1 (1:1000, Invitrogen), occludin (1:1000, Invitrogen), claudin-1 (1:1000, Invitrogen), Atg5 (1:1000, Sigma), p62 (1:1000, Sigma), LC3 (1:1000, Sigma), NLRP3 (1:1000, Adipogen), ASC (1:1000, Cell Signaling), caspase 1 (1:1000, Cell Signaling), cleaved caspase 1 (1:1000, Cell Signaling), IL-1β (1:1000, Cell Signaling), cleaved IL-1β (1:1000, Cell Signaling), IL-18 (1:1000, Cell Signaling) and β-actin (1:5000, Sigma) overnight at 4°C. After wash with TBST, membranes were incubated with appropriate peroxidase-conjugated secondary antibodies (KPL) at room temperature for 60 min. The blots were visualized with an enhanced chemiluminescence detection kit (GE Healthcare, Buckinghamshire, UK). The chemiluminescence signal was captured by a ChemiDox XRS system (Bio-Rad). The densities of the bands were quantified with Quantity One Image software (Bio-Rad).

### SCFAs analysis via HS-GC/MS

Fresh ileocecal fecal matters were frozen immediately and stored at -80°C until processing. Weighed fecal matters were put into a 20ml headspace sampling bottle, followed by the addition of 0.95ml 6% H_3_PO_4_ and 0.05ml 2.49 mmol/L of 2-ethylbutyric acid (the internal standard, diluted by 6% H_3_PO_4_), and vibration for 1 min. Then samples were injected into the gas chromatography-mass spectrometry system (5975A/7890C Agilent) for analysis. Ultrapure Helium (1.0 mL/min) was used as the carrier gas. Headspace was maintained at 80°Cwith incubation time of 30min. The gas chromatography (DB-FFAP fused-silica capillary column: 30 m ×0. 25 mm ×0. 25 μm Agilent) stepwise thermal conditions were as follows: 50°C for 1 min, 10°C /min until 250°C for 2 min; injector temperature was 250°C, with split ratio of 5:1. Mass detector system was operated in electron impact ionization at 70 eV, set to scan mode for m/z 33–200, and in single ion monitoring (SIM) mode. Temperatures of the transfer line (interface), and source were maintained at 280°C and 250°C, respectively. The monitored ions were m/z 60 and 43.1 for acetic acid, m/z 74.1 and 28.1 for propionic acid, m/z 60 and 73.1 for butyric acid, m/z 60 and 87.1 for isovaleric acid, m/z 43.1 and 73.1 for isobutyric acid, and m/z 88.1 and 73.1 for 2-ethylbutyric. Data acquisition and analysis were done with Saturn GC/MS workstation software (Agilent).

### DNA extraction

Fresh ileocecal fecal matters were frozen immediately after collected and stored at -80°C until DNA isolation. Microbial DNA was extracted with the QIAamp® Fast DNA Stool Mini Kit (Qiagen, Valencia, CA) in accordance with manufacturer’s protocols, and then stored at -80°C for further processing.

### PCR amplification

The V3-V4 region of bacterial 16S rDNA gene was amplified using primers 338F (5’-ACTCCTACGGGAGGCAGCAG-3’) and 806R (5’-GGACTACHVGGGTWTCTAAT-3’). The PCR reactions (95°C for 3 min, followed by 27 cycles at 95°C for 30s, 55°C for 30s, and 72°C for 45s and a final extension at 72°C for 10 min) were performed in 20μL mixture containing 4μL of 5× FastPfu Buffer, 2μL of 2.5 mmol/L dNTPs, 0.8μL of primers (5μmol/L), 0.4μL of FastPfu Polymerase, and 10 ng of template DNA. Illumina MiSeq sequencing amplicons were extracted from 2% agarose gels, purified using AxyPrep DNA Gel Extraction Kit (Axygen Biosciences, Union City, CA, USA) according to manufacturer’s instructions, and quantified using QuantiFluor™-ST (Promega, USA). Purified amplicons were pooled in equimolar and paired-end sequenced (2 × 250) on an Illumina MiSeq platform according to the standard protocols. The raw reads were deposited into the NCBI Sequence Read Archive (SRA) database.

### Processing of sequencing data

The raw fastq files were demultiplexed, quality-filtered using QIIME (version 1.9.1) with the following criteria: (i) the 300 bp reads were truncated at any site receiving an average quality score <20 over a 50 bp sliding window, discarding truncated reads that were shorter than 50bp. (ii) exact barcode matching, 2 nucleotide mismatch in primer matching, reads containing ambiguous characters were removed. (iii) only sequences that overlap longer than 10 bp were assembled according to their overlap sequence. The reads which could not be assembled were discarded. Operational Units (OTUs) were clustered with 97% similarity cutoff using UPARSE (version 7.1 http://drive5.com/uparse/) and chimeric sequences were identified and removed using UCHIME. The taxonomy of each 16S rRNA gene sequence was analyzed by RDP Classifier (http://rdp.cme.msu.edu/) against the silva (SSU123)16S rRNA database using confidence threshold of 70%.

### Statistical analysis

Data are presented as means ± SEM. For data analysis, one-way ANOVA was performed by SPSS 13.0 statistical software. A *p* value of <0.05 was considered as minimum level of statistical significance in all cases. All reported significant levels represent two-tailed *p* values.

## Results

### Antibiotics alters the diversity and composition of intestinal microbiota

**Fig 1:**
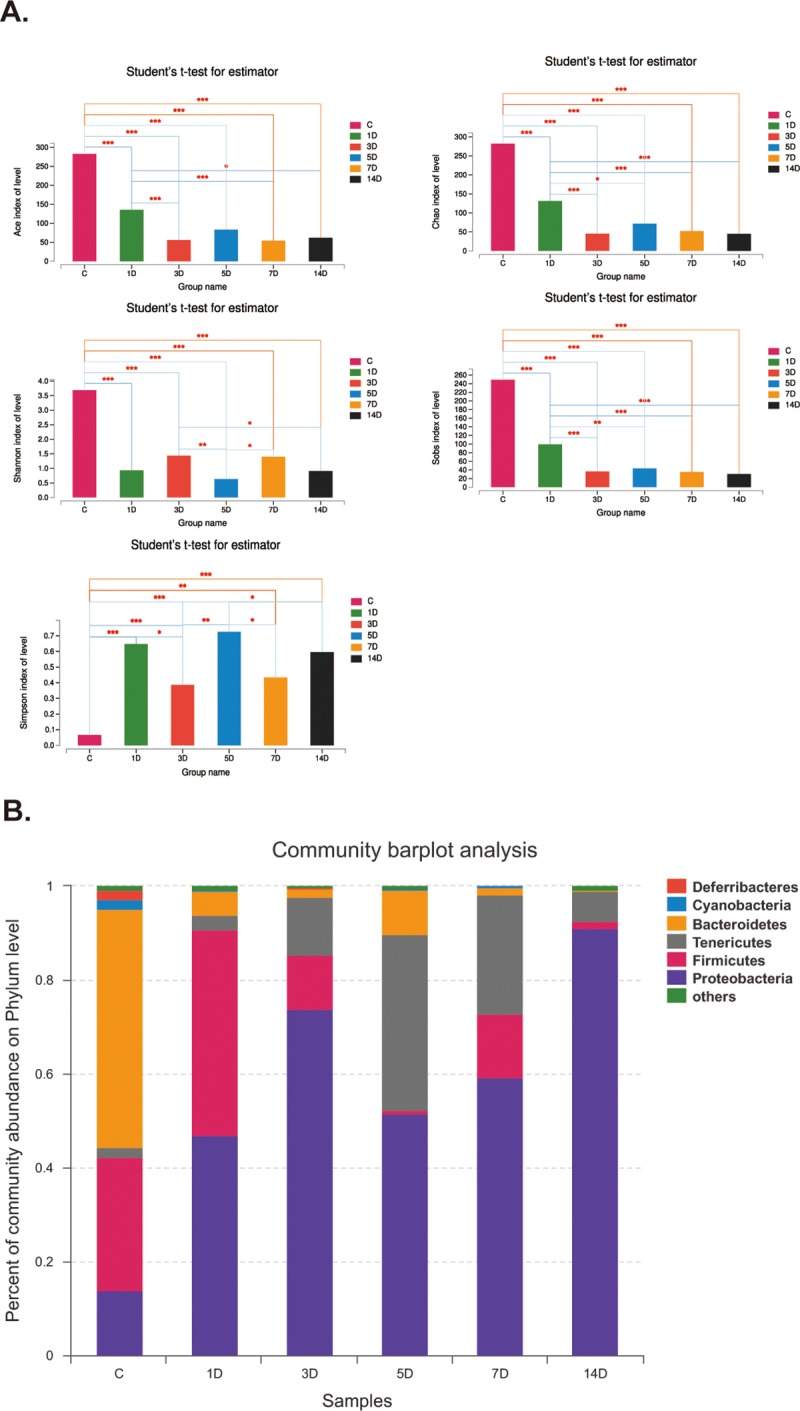
Antibiotics alters diversity and composition of intestinal microbiota. A. Antibiotics statistically reduced Ace, Chao, Shannon and Sobs, which are the indices of intestinal microbiota. *p<0.05, ***p<0.001 compared with control. B. Percent of community abundance on phylum level. Antibiotics significantly increased the percentage of Proteobacteria.

To figure out the alterations of intestinal microbiota after antibiotic treatment, we evaluated the composition and diversity through an Illumina high-throughput sequencing technique. We generated a dataset consisting of 1698557 filtered high-quality and classifiable 16S rDNA gene sequences, and the average number of sequences obtained for each individual was 36139.5 (range: from 30068 to 44125). All sequences were clustered with representative sequences, and a 97% sequence identification cut-off was used. Obtained total number of operational taxonomic units (OTUs) was 474. As shown in Fig 1A, all diversity indexes including Ace, Chao, Shannon and Sobs were significantly decreased after antibiotics treatment, except Shannon which was not statistical different among 3 day, 5 day, 7 day and 14 day groups. However, Simpson was evidently increased without statistical discrepancy among 3 day, 7 day and 14 day groups. Furthermore, bacterial composition structure was analyzed. As shown in Fig 1B, on phylum level, *Bacteroidetes*, *Firmicutes* and *Proteobateria* were the dominant bacteria in control mice. However, in antibiotics-treated mice, the proportions of *Proteobateria* and *Tennericutes* were obviously increased, whereas the percentages of *Bacteroidetes* and *Firmicutes* were evidently decreased. Thus, broad-spectrum antibiotics remarkably alter the diversity and composition of intestinal microbiota in healthy mice.

### NMDS and LEfSe analysis of intestinal microbiota composition after antibiotics treatment

**Fig 2:**
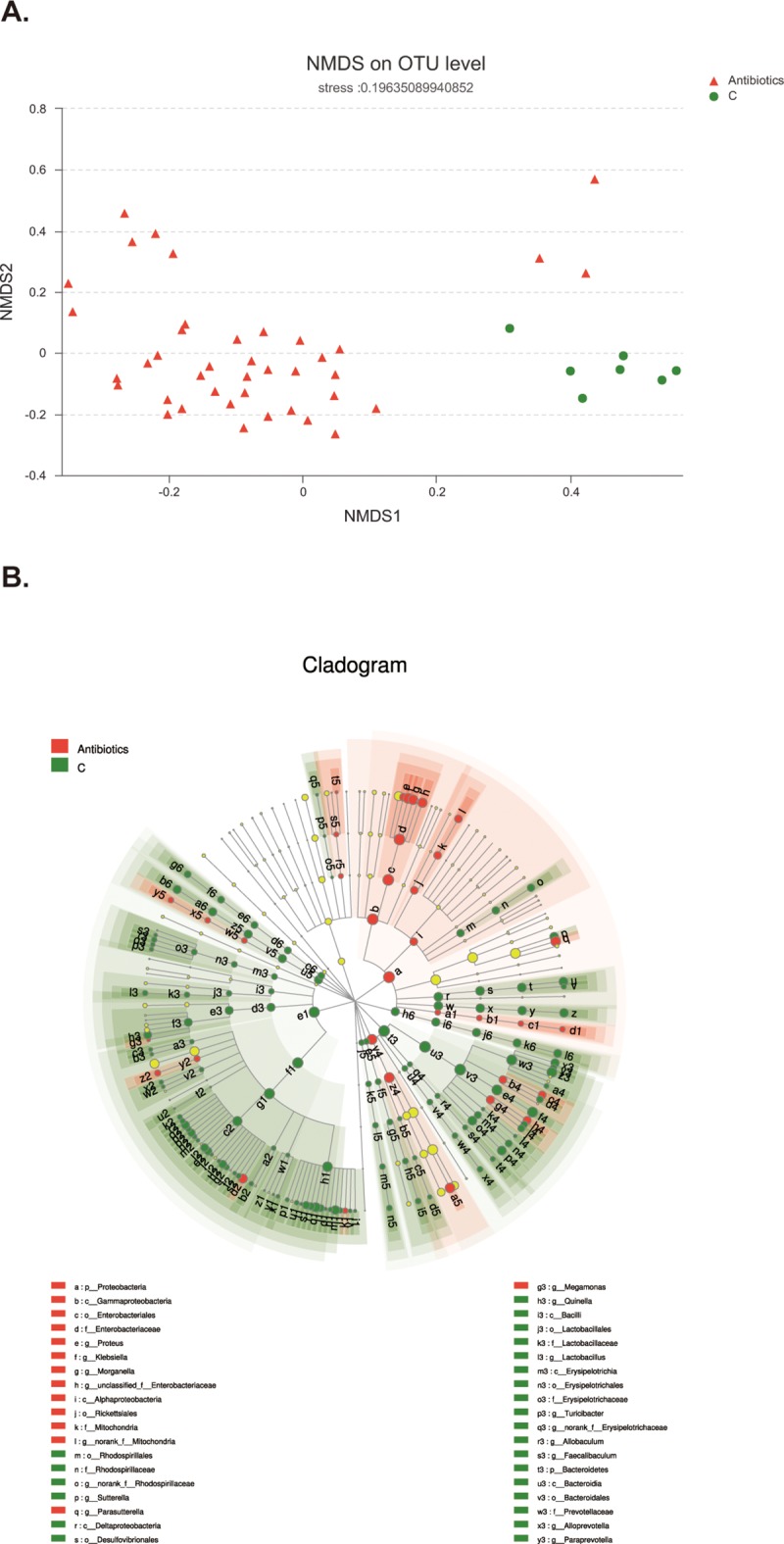
NMDS and LEfSe analysis of intestinal microbiota composition after antibiotics treatment. A. The green dots, representing the distributions of samples in control group, mainly distribute at the lower right area. The red triangles, standing for samples of antibiotics group, locate at the left area. B. Green symbolizes the differential bacteria in control group, and red stands for bacteria with significant difference in antibiotics group. Species with in-apparent variation are represented by yellow dot. The circle is segmented into five layers, respectively representing phylum, class, order, family, and genus levels from the inside out.

Based on the above results, we expected to further figure out the different species after antibiotics treatment, therefore, we re-divided these 47 samples into two groups, i.e. control group (C-1,C-2,C-3,C-4,C-5,C-6,C-7), antibiotics group containing allsamples treated by antibiotics for different durations. Then, we employed non-metric multidimensional scaling (NMDS) to reveal the dissimilarities in microbial composition on OTU level. As shown in Fig 2A, control group samples (green dots) accumulated on the lower right area, whereas most of antibiotics group samples (red triangles) were distributed on the lower left side, implying considerable discrepancy between two groups. Next, linear discriminant analysis effect size (LEfSe) method was employed to figure out the exact species with significant difference between two groups. As illustrated in Fig 2B, there were almost 168 species with significant variations. Specifically, *p_Proteobacteria*, *c_Gammaproteobacteria*, *o_Enterobacteriales* and other 32 species had statistical difference in antibiotics group. In addition, species including *p_Bacteroidates*, *f_Prevotellaceae*, *f_Lachnospiraceae* and other 130 species were the noticeable varied bacteria in control group. Taken together, broad-spectrum antibiotics are capable of disrupting intestinal microbiota composition.

### Potential biomarkers for antibiotics treatment

**Fig 3:**
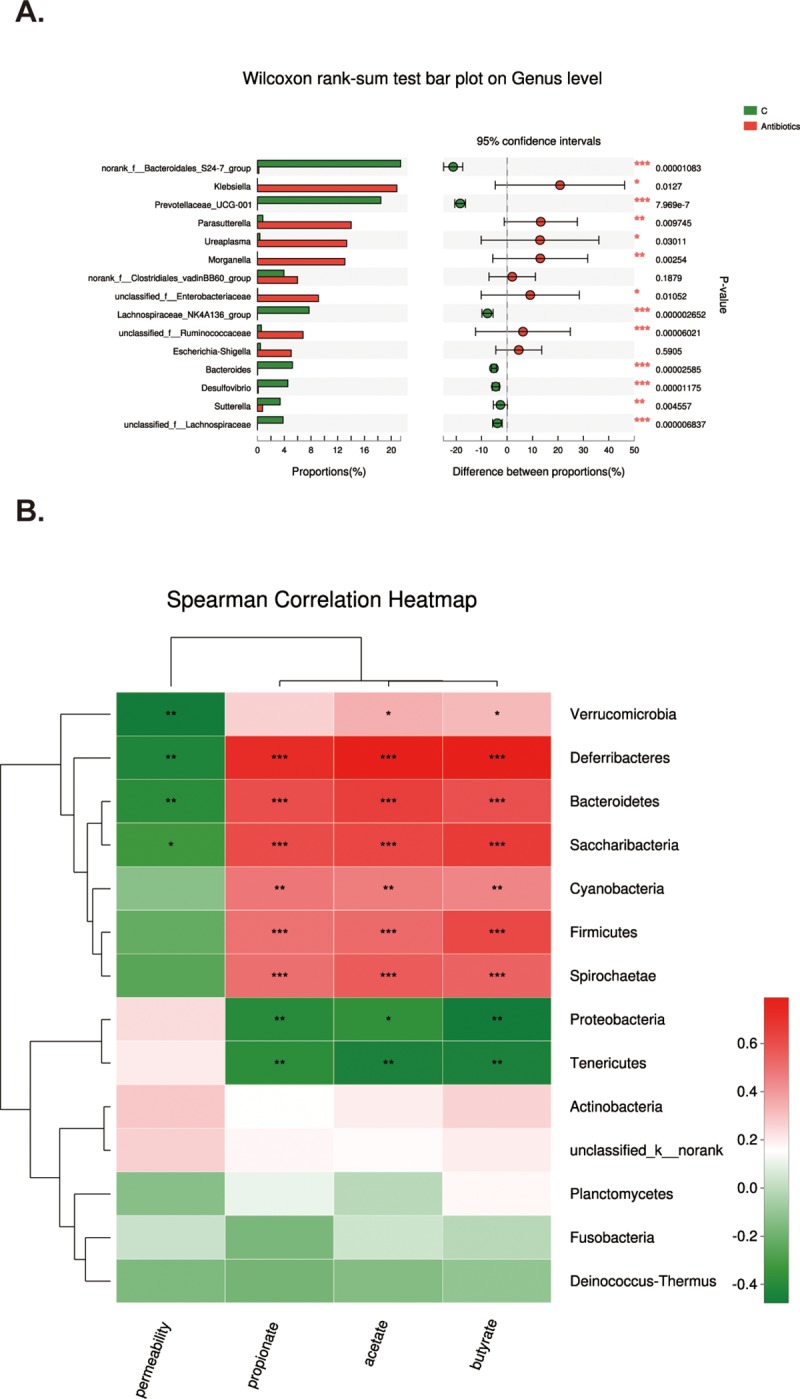
Potential biomarkers and correlation analysis. A. Wilcoxon rank sum test analysis to figure out the potential biomarkers. X axis is percentage value of specific species abundance, and Y axis represents species name at various classification levels. *p<0.05, **p<0.01, ***p<0.001 when compared with control group. B. X axis is environmental factors including intestinal permeability, acetate, propionate, and butyrate. Y axis represents specific species on phylum level. Spearman correlation coefficients are represented by color ranging from blue, negative correlation (-0.4), to red, positive correlation (0.6). *p<0.05, **p<0.01, ***p<0.001 when compared with control group.

Based on NMDS and LEfSe analysis, we further utilized wilcoxon rank sum test to figure out potential bio-markers for antibiotics treatment. As shown in Fig 3A, the specific differential bacteria in control group were *norank_f_Bacteroidales_S24-7_group* (*p*<0.001), *Prevotellaceae_UCG-001* (*p*<0.001), *Lachnospiraceae_NK4A136_group* (*p*<0.001), *Bacteroides* (*p*<0.001), *Desulfocibrio* (*p*<0.001), *Sutterella* (*p*<0.001) and *unclassified_f_Lachnospiraceae* (*p*<0.001). However, the specific differential species in antibiotics group were *Klebsiella* (*p*<0.001), *Parasutterella* (*p* = 0.009), *Ureaplasma* (*p* = 0.03), *Morganella* (*p* = 0.002), *unclassified_f_Enterobacteriaceae* (*p* = 0.01), *unclassified_f_Ruminococcaceae* (*p*<0.001). Thus, broad-spectrum antibiotics could produce some potential biomarkers which may have important biological significance.

### Correlative analysis between intestinal microbiota community and several environment factors

To address the correlation between specific bacteria species (top 50 species in abundance) and environmental factors including SCFAs’ concentrations and intestinal permeability, we employed Spearman correlation analysis on Phylum level. As shown in Fig 3B, intestinal permeability was negatively correlated with *Verrucomicrobia*, *Deferribacteres*, *Bacteroidetes* and *Saccharibacteria*. All SCFAs investigated in this study, including acetate, propionate and butyrate, were positively correlated with *Deferribacteres*, *Bacteroidetes*, *Saccharibacteria*, *Cyanobacteria*, *Firmicutes* and *Spirochaetae*, and also negatively correlated with *Proteobacteria* and *Tenericutes*. Besides, both acetate and butyrate were positively correlated with *Verrucomicrobia*. In brief, there are so many species show correlation with SCFAs, acting as the possible explanations for several phenomenon which will be described below.

### Functional prediction of intestinal microbiota community after antibiotics treatment

**Fig 4:**
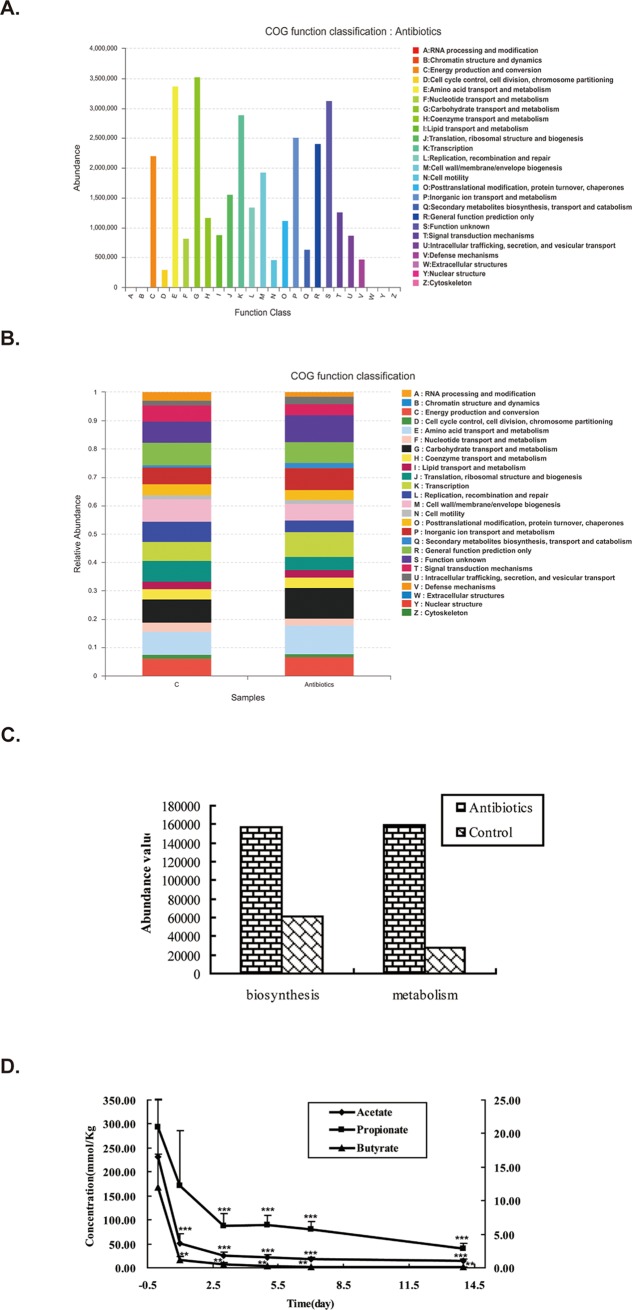
Functional prediction of intestinal microbiota community. A. COG function classification of antibiotics group. X axis represents function class, and Y axis stands for abundance. B. Comparison of COG function between control and antibiotics group. X axis represents samples and Y axis stands for relative abundance. C. Comparison of abundance related to short chain fatty acids biosynthesis and metabolism. X axis represents different functions and Y axis stands for abundance value. D. Antibiotics decreased SCFAs concentrations. Concentrations of SCFAs (acetate, propionate and butyrate) in ileocecal feces were assessed after antibiotics treatment. Antibiotics remarkably decreased the concentrations of all SCFAs. Data represent the mean±SEM. *p<0.05, **p<0.01, ***p<0.001 compared with control.

Furthermore, we conducted function analysis of intestinal microbiota in order to figure out the possible functions of intestinal microbiota after antibiotics treatment. We employed cluster of orthologous groupe (COG) and Kyoto Encyclopedia of Genes and Genomes (KEGG) to analyze the possible functions and metabolic pathways respectively. As shown in Fig 4A, after antibiotics treatment, the principal functions of species were associated with transport and metabolism of both amino acids and carbohydrates, production and conversion of energy, and others. However, as illustrated in Fig 4B, when compared with that of control group, the relative abundance of species whose functions are associated with transcription, the transport and metabolism of amino acid, carbohydrate and inorganic ion were remarkably increased, whereas the relative abundance of species whose functions are related to cell membrane biogenesis, defense mechanisms, replication, recombination and repair were markedly decreased. Furthermore, as illustrated in Fig 4C, the abundance of species which function in biosynthesis increased to 2.57 folds of control. Meanwhile, the abundance of species associated with fatty acid metabolism also augmented to 5.75 folds of control. Taken together, antibiotics induce significant alterations in intestinal microbiota homeostasis.

### Antibiotics decreases SCFAs concentrations

It has been demonstrated that SCFAs are the principal byproducts of intestinal microbiota [19]. Therefore, we next investigated whether antibiotics alter intestinal SCFAs concentrations in healthy mice. As shown in Fig 4D, concentrations of all detected SCFAs, including acetate, propionate and butyrate, were significantly decreased after antibiotics treatment, especially on 14th day. Thus, broad-spectrum antibiotics could remarkably down-regulate intestinal SCFAs concentrations, which might be the potential contributor to pathological alterations induced by antibiotics.

### Antibiotics impairs intestinal tight junction barrier

**Fig 5:**
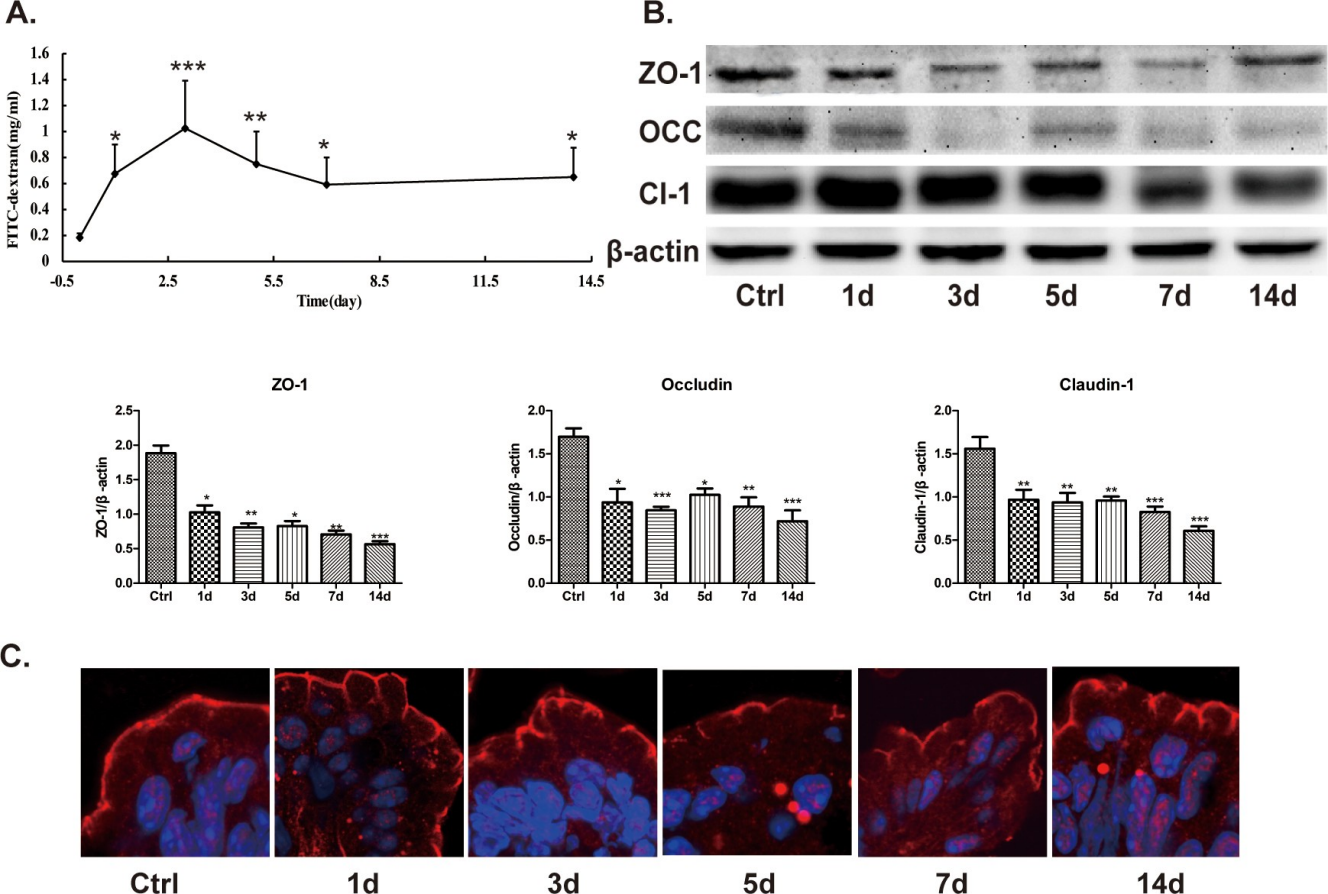
Antibiotics impairs intestinal barrier function. A. Intestinal permeability to 4.4 kDa FITC-dextran was significantly increased after antibiotics treatment. Data represent the mean±SEM (n = 6). *p<0.05, **p<0.01, ***p<0.001 compared with control. B. Antibiotics significantly decreased proteins expressions of ZO-1, occludin and claudin-1. Data represent the mean±SEM (n = 6). *p<0.05, **p<0.01, ***p<0.001 compared with control. OCC: occludin, CL-1: claudin-1. C. ZO-1 (red) was mainly localized to the epithelial tight junctions in the ileum of control mice. In contrast, antibiotics disrupted the normal arrangement of ZO-1 at the apical junctions. Data are representative of three independent experiments. Scar bar = 10.0μm.

It has been reported that intestinal microbiota and its derived SCFAs are crucial to intestinal barrier integrity [20, 21]. Thus, we further investigated the effect of antibiotics treatment on intestinal tight junction barrier in healthy mice. As shown in Fig 5A, when compared with that of control (0 day), intestinal permeability to 4.4kDa FITC-dextran was significantly increased after antibiotics treatment. Meanwhile, we evaluated the expressions of ZO-1, occludin, and claudin-1, which are important members of tight junction proteins. As illustrated in Fig 5B, when compared with those of control, the expressions of ZO-1, occludin and claudin-1 were gradually decreased after antibiotics treatment. Next, we assessed the morphological changes of ZO-1 by immunofluorescent assay. As shown in Fig 5C, in ileum of control mice, ZO-1 was localized to epithelial tight junction, which was appreciated as red spots lying at the apical compartment of cell-cell junctions. In contrast, this ordered appearance of ZO-1 was disrupted in ileum of antibiotics treated mice. Taken together, it is indicated that broad-spectrum antibiotics impairs intestinal tight junction barrier, as evidenced by increased intestinal paracellular permeability, reduced expressions of tight junction proteins and disrupted localization of ZO-1.

### Antibiotics activates NLRP3 inflammasome

**Fig 6:**
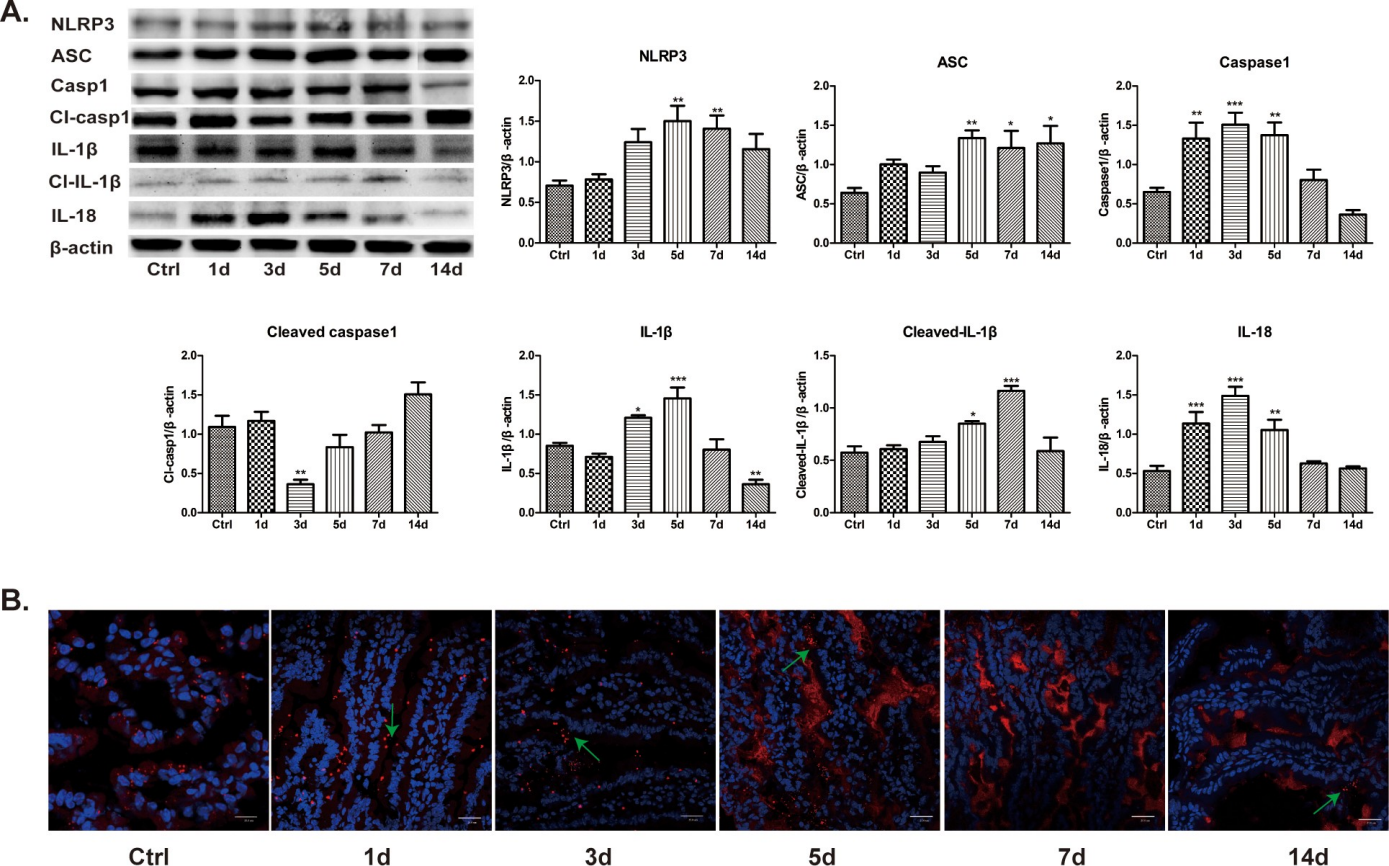
Antibiotics activates NLRP3 inflammasome. A. After antibiotics treatment, protein expressions of NLRP3, ASC, cleaved caspase-1, cleaved IL-1β and IL-18 increased significantly. But expressions of caspase-1 and IL-1β decreased gradually. Data represent the mean±SEM (n = 6). *p<0.05, **p<0.01, ***p<0.001 compared with control. B. Antibiotics remarkably augmented the amount of ASC protein (red punctate). Scale bar = 25.0μm.

It has been reported that microbiota interact with NLRP3 inflammasome in intestine [22, 23], and that NLRP3 inflammasome could remodel intestinal microbiota [24]. Therefore, we next determined whether the intestinal microbiota dysbiosis induced by antibiotics was accompanied by the activation of NLRP3 inflammasome. Once activated, NLRP3 recruited the adaptor protein ASC and pro-caspase-1 to form a multiprotein complex termed inflammasome, and then stimulates the secretions of IL-1β and IL-18^[^^25^^]^. As illustrated in Fig 6A, expressions of NLRP3, ASC, caspase 1, cleaved caspase 1, IL-1β, cleaved IL-1β and IL-18 were significantly increased at different time points after antibiotics treatment, as compared with control. Consistent with these, immunofluorescence assay also revealed the amounts of red punctate, representing ASC protein, were evidently increased after antibiotics treatment, as shown in Fig 6B. Taken together, the results indicate that NLRP3 inflammasome is markedly activated by antibiotics treatment, which may contribute to antibiotics-induced intestinal barrier dysfunction.

### Antibiotics activates autophagy

**Fig 7:**
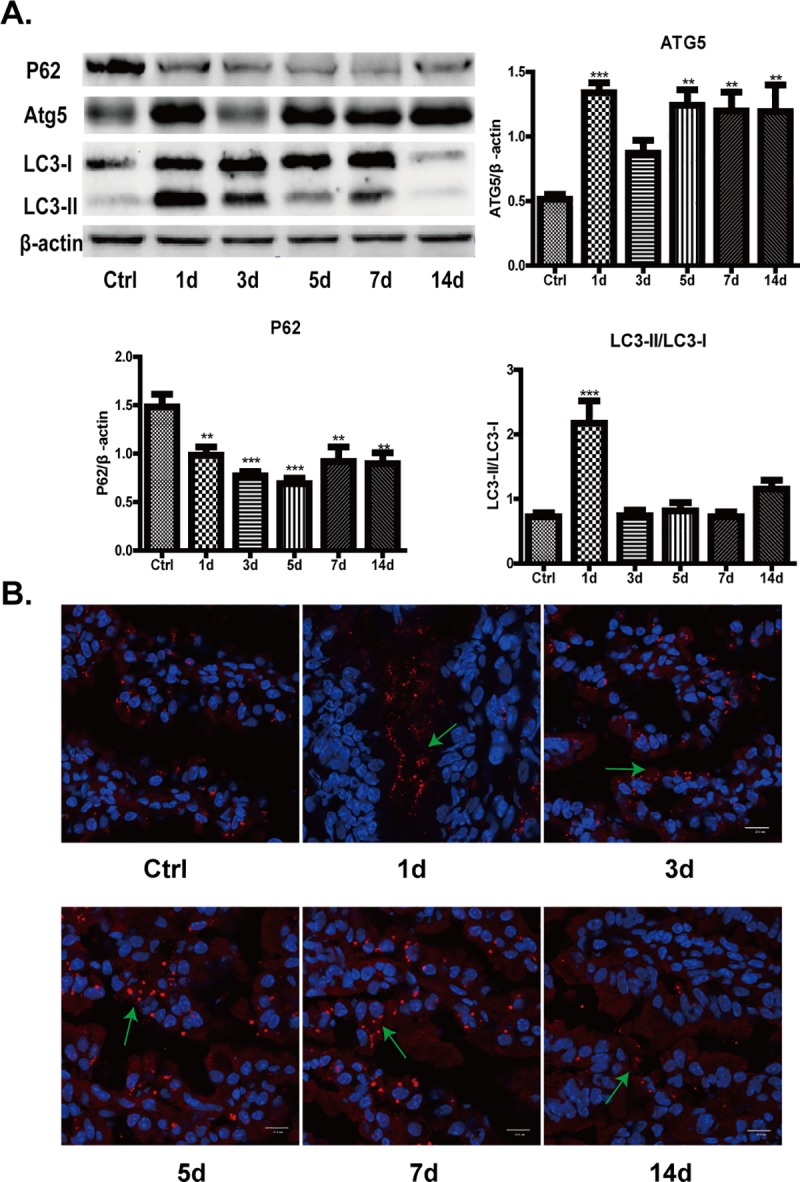
Antibiotics activates autophagy. A. Antibiotics increased the expressions of Atg5 and ratio of LC3-II/LC3-I, but decreased P62 expression. Data represent the mean±SEM (n = 6). **p<0.01, ***p<0.001 compared with control. B. Antibiotics increased the amount of LC3B (red punctate). Scale bar = 25.0μm.

Intestinal microbiota such as *Fusobacterium nucleatum* could regulate autophagy [26], therefore, we attempted to investigate whether autophagy was activated by intestinal microbiota dysbiosis caused by antibiotics. Microtubule-associated protein light chain (LC3), which is lipidated and incorporated into autophagosomal membrane, is widely used as a marker for autophagy [27]. Besides, autophagy substrates including P62 and Atg5 are also recognized as the markers for autophagy, because P62 is an adaptor protein which binds to LC3, and Atg5 facilitates the phosphatidylethanolamine conjunction of LC3^[^^28^^,^ ^29^^]^. As shown in Fig 7A, both LC3-II/LC3-I ratio and Atg5 expression were remarkably increased after antibiotics treatment. Accordingly, P62 expression was evidently reduced. In parallel to these, fluorescent assay demonstrated that the amount of red punctate representing LC3B augmented markedly at different time points after antibiotics treatment, as revealed in Fig 7B. These results indicate that autophagy is activated by intestinal microbiota dysbiosis induced by antibiotics, which may contribute to the occurrence of intestinal barrier dysfunction in antibiotics treated mice.

## Discussion

Gastrointestinal tract is a complex micro-ecosystem, in which inhabits a huge and varied microbial community consisting of bacteria, archaea, viruses and various unknown eukaryotes. Intestinal microbiota alterations are associated with various diseases. It has been reported that intestinal microbiota community could be obviously modified by various factors, such as antibiotics, probiotics and prebiotics, and may be one necessary pathologic mechanism for some intestinal diseases including inflammatory bowel disease [30]. Here, we demonstrate that antibiotics significantly reduce intestinal microbiota diversity indexes, including Ace, Shannon, Sobs and Chao. Meanwhile, antibiotics increase *Proteobateria* and *Tennericutes*, but decrease *Bacteroidetes* and *Firmicutes*. In accordance with our findings, previous studies have reported that oral vancomycin reduces fecal microbial diversity with a decrease of gram-positive bacteria (mainly *Firmicutes*) and a compensatory increase of gram-negative bacteria (mainly *Proteobacteria*) [31, 32]. We also show that the potential biomarkers after antibiotics treatment might be *Klebsiella*, *Parasutterella*, *Morganella*, *unclassified_f_Enterobacteriaceae*, *Ureaplasma* and *unclassified_f_Ruminococcaceae*, majority of which belong to *Proteobateria* phylum. In line with our findings, *Proteobateria* has been reported to be a potential diagnostic signature of intestinal microbiota dysbiosis [32]. In addition, antibiotics increase relative abundance of species whose functions are associated with the transport and metabolism of amino acids and carbohydrates, but reduce the species whose functions are related to cell membrane biogenesis, defense mechanisms, replication, recombination and repair. The abundance of species associated with the biosynthesis and metabolism of fatty acids were remarkably increased by antibiotics. Thus, antibiotics induce intestinal microbiota dysbiosis and alter intestinal microbiota homeostasis.

In this study, we also show that altered intestinal microbiota community is associated with some environmental factors including increased intestinal permeability and decreased SCFAs’ (acetate, propionate and butyrate) concentrations. Specifically, intestinal permeability was negatively correlated with *Verrucomicrobia*, *Deferribacteres*, *Bacteroidetes* and *Saccharibacteria*. SCFAs were negatively correlated with *Proteobacteria* and *Tenericutes*, but positively correlated with *Bacteroidetes* and *Firmicutes*. In line with our results, other investigators have documented that SCFAs are positively correlated with *Firmicutes* (*Bacillales*, *Sporolactobacillus and Lactobacillus*) [33]. Moreover, it has been revealed that while *Proteobateria* decreased, butyrate-producing bacteria (*Lactobacillus* and *Prevotella*) increased in fermented soybean meal fed piglets, implying that *Proteobateria* and butyrate concentration have negative correlation [34].

Antibiotics have been well reported to induce intestinal microbiota dysbiosis [14,35,36]. However, consensus of their effects on intestinal barrier are deficient, particularly regarding tight junction barrier. In this study, we, for the first time, demonstrate that antibiotics increase intestinal paracellular permeability, decrease tight junction proteins’ expressions, and alter ZO-1 morphology, suggesting that antibiotics induce intestinal tight junction barrier dysfunction *in vivo*. In accordance with our findings, previous studies have demonstrated that antibiotics, such as Clamoxyl and clindamycin, affect intestinal barrier function through disrupting intestinal microbiota or other mechanisms in animals [37–39]. Furthermore, antibiotics are detrimental to intestinal barrier in patients and mice [40]. Taken together, it is suggested that antibiotics are able to disrupt intestinal tight junction barrier *in vivo*. In addition, the increased *Firmicutes/Bacteroidetes* ratio was accompanied by improved intestinal barrier function in *P*. *americana* ethanol extract treated ulcerative colitis rats [41]. However, the reduced *Firmicutes/Bacteroidetes* ratio was along with augmented tight junction proteins in diammonium glycyrrhizinate-treated nonalcoholic fatty liver disease in mice [42]. Thus, variation of *Firmicutes* and *Bacteroidetes* could be closely associated with intestinal tight junction barrier. Therefore, antibiotics-induced intestinal barrier dysfunction might be tightly related to the variation of *Firmicutes* and *Bacteroidetes*.

Given that intestinal microbiota dysbiosis contributes to antibiotics-induced intestinal tight junction barrier disruption, however, underlying mechanisms are still unclear. It has been well recognized that NLRP3, the best characterized member of the NLRP sub-family, recruits the adaptor protein ASC and pro-caspase-1 to form a multiprotein complex termed inflammasome, triggering IL-1β and IL-18 processing and release [25,43]. Therefore, NLRP3 inflammasome plays an important role in regulating intestinal homeostasis [43]. In addition, intestinal microbiota has been reported to interact with NLRP3 inflammasome [22,23]. In this study, we demonstrate that antibiotics-induced intestinal microbiota dysbiosis and SCFAs reduction are accompanied by NLRP3 inflammasome activation, as demonstrated by increased expressions of NLRP3, ASC, caspase 1, cleaved caspase 1, IL-1β, cleaved IL-1β and IL-18. In accordance with our findings, a previous study has revealed that intestinal microbiota deficiency and decreased SCFAs’ productions are necessary for NLRP3 inflammasome activation and IL-1β production [44], suggesting that intestinal microbiota dysbiosis is critical for NLRP3 inflammasome activation. However, it has been reported that intestinal microbiota is remodeled by hyperactive NLRP3 inflammasome and then maintain homeostasis [24], implying that both NLRP3 inflammasome and intestinal microbiota have interaction with each other. The NLRP3 inflammasome activation may result in intestinal barrier dysfunction. A recent study has demonstrated that activated NLRP3 inflammasome by microbial products induces the synthesis of proinflammatory cytokines, decreases expressions of ZO-1 and E-cadherin, and increases permeability to FITC-dextran, leading to the epithelial barrier dysfunction in cholangiocytes [45]. Similarly, another recent study has revealed that titanium dioxide-triggered NLRP3 inflammasome activation increases the permeability to FITC-dextran in Caco-2 monolayers [46]. In consistent with these studies, our latest investigation has shown that induction of NLRP3 inflammasome activation with both lipopolysaccharide and ATP decreases transepithelial electrical resistance, reduces the expressions of ZO-1, occludin and claudin-1, increases expression of claudin-2, and causes the re-localization of ZO-1 and occludin in Caco-2 monolayers [47]. Therefore, activation of NLRP3 inflammasome may contribute tointestinal tight junction barrier dysfunction.

It has been recognized that there is an interplay between NLRP3 inflammasome and autophagy [48,49], however, this interaction is far more complex than it has been articulated. Autophagy, an intracellular degradation pathway and an essential cell survival mechanism, refers to the engulfment and processing of cellular proteins, including damaged organelles, long-lived and mis-folded proteins. Autophagy has been reported to play an important role in diverse processes such as metabolic stress and inflammatory bowel diseases [27, 50]. In this study, we show that antibiotics activate autophagy, as evidenced by decreased P62 expression and increased LC3-II/LC3-I ratio and Atg5 expression. Although autophagy activation has been reported to be closely associated with intestinal barrier dysfunction [16,51,52], the precise role of autophagy activation in regulating barrier function is still controversial. Some studies have demonstrated that autophagy maintained intestinal or endothelial barrier [53,54]. In contrast, it has been revealed that autophagy disrupts endothelial barrier, and autophagy inhibition protects against endothelial barrier dysfunction by suppressing cadherin disassembly and actin stress fiber formation [55], indicating autophagy induces endothelial barrier dysfunction. Consistent with this finding, our latest *in vitro* study has revealed that autophagy decreases transepithelial electrical resistance and expressions of ZO-1, occludin and claudin-1, increases expression of claudin-2, and disrupts the morphology of ZO-1 and occludin in Caco-2 monolayers [47]. Thus, autophagy may be involved in antibiotics-induced intestinal tight junction barrier dysfunction.

In conclusion, the principle finding of this study is that antibiotics could severely destroy intestinal microbiota community including composition and function, accompanying reduced SCFAs concentration. More importantly, antibiotics induce intestinal tight junction barrier dysfunction. Furthermore, antibiotics activates NLRP3 inflammasome and autophagy in intestine. These findings provide a new insight into the association between antibiotics-induced intestinal microbiota dysbiosis and intestinal tight junction barrier dysfunction, in which activated NLRP3 inflammasome and autophagy may be involved. However, the precise role of *Proteobateria* and *Tennericutes* in intestinal barrier dysfunction, and concrete relationship between NLRP3 inflammasome and autophagy need to be further determined.

## Supporting information

S1 File Dataset. (XLSX)
